# Diffusion Tensor and Volumetric Magnetic Resonance Measures as Biomarkers of Brain Damage in a Small Animal Model of HIV

**DOI:** 10.1371/journal.pone.0105752

**Published:** 2014-08-21

**Authors:** Margaret R. Lentz, Kristin L. Peterson, Wael G. Ibrahim, Dianne E. Lee, Joelle Sarlls, Martin J. Lizak, Dragan Maric, William C. Reid, Dima A. Hammoud

**Affiliations:** 1 Center for Infectious Disease Imaging (CIDI), Radiology and Imaging Sciences, Clinical Center, National Institutes of Health (NIH), Bethesda, Maryland, United States of America; 2 National Institute of Child Health and Human Development (NICHD), NIH, Bethesda, Maryland, United States of America; 3 Magnetic Resonance Imaging Research Facility (NMRF), National Institute of Neurological Disorders and Stroke (NINDS), NIH, Bethesda, Maryland, United States of America; 4 Division of Intermural Research (DIR), National Institute of Neurological Disorders and Stroke (NINDS), NIH, Bethesda, Maryland, United States of America; University of Texas Southwestern Medical Center, United States of America

## Abstract

**Background:**

There are currently no widely accepted neuro-HIV small animal models. We wanted to validate the HIV-1 Transgenic rat (Tg) as an appropriate neuro-HIV model and then establish in vivo imaging biomarkers of neuropathology, within this model, using MR structural and diffusion tensor imaging (DTI).

**Methods:**

Young and middle-aged Tg and control rats were imaged using MRI. A subset of middle-aged animals underwent longitudinal repeat imaging six months later. Total brain volume (TBV), ventricular volume (VV) and parenchymal volume (PV = TBV–VV) were measured. Fractional anisotropy (FA) and mean diffusivity (MD) values of the corpus callosum (CC) were calculated from DTI data.

**Results:**

TBV and PV were smaller in Tg compared to control rats in young and middle-aged cohorts (*p*<0.0001). VV increased significantly (*p* = 0.005) over time in the longitudinal Tg cohort. There were lower FA (*p*<0.002) and higher MD (*p*<0.003) values in the CC of middle-aged Tg rats compared to age-matched controls. Longitudinally, MD significantly decreased over time in Tg rats (*p*<0.03) while it did not change significantly in the control cohort over the same period of time (*p*>0.05).

**Conclusions:**

We detected brain volume loss in the Tg rat, probably due to astrocytic dysfunction/loss, loss of structural/axonal matrix and striatal neuronal loss as suggested by immunofluorescence. Increased MD and decreased FA in the CC probably reflect microstructural differences between the Tg and Control rats which could include increased extracellular space between white matter tracts, demyelination and axonal degeneration, among other pathologies. We believe that the Tg rat is an adequate model of neuropathology in HIV and that volumetric MR and DTI measures can be potentially used as biomarkers of disease progression.

## Introduction

Even in the era of anti-retroviral therapy (ART), 30–50% of HIV-positive (HIV+) subjects on a successful treatment regimen will develop some degree of cognitive impairment [1], [2]. The exact pathophysiology of neurological damage in HIV is not well understood [3], and there is lack of accurate *in vivo* markers of glial response or neuronal injury in this disease. Advanced imaging modalities, combined with suitable animal models and *in vitro* molecular biology techniques, can provide insight into the pathophysiology underlying HIV-associated cognitive impairment and provide means for preclinical evaluation of disease-modifying approaches.

Over the past few decades, both structural MRI and diffusion tensor imaging (DTI) studies have been used in the evaluation of HIV+ subjects [4]–[7]. DTI is a magnetic resonance imaging (MRI) technique that allows for non-invasive measurement of water diffusion characteristics in brain tissues, which can then be exploited to determine fiber tract orientation [8]–[10]. In a free fluid state, water diffusion is usually uniform in all directions, or “isotropic”. In biologic tissues, on the other hand, water diffusion is comparatively non-uniform and more directional due to the presence of various physiological obstacles such as macromolecules, cell membranes, and fiber tracts. This is referred to as anisotropic motion. Anisotropy is especially high in white matter due to the presence of bundled fiber tracts which impede water diffusion perpendicular to their long axis while at the same time allowing water diffusion parallel to the same axis [11]–[14]. Using DTI, the characteristics of water diffusion in a voxel of tissue can be mathematically represented by a three-dimensional diffusion ellipsoid tensor. This ellipsoid is characterized by the magnitude of its three axes or “eigenvalues” (λ1, λ2, and λ3), and their orientations in space or “eigenvectors” (ε1, ε2, and ε3). The primary (longitudinal) eigenvector thus provides information about the direction of maximum diffusion within a voxel and is the basis for three-dimensional fiber tracking. From this ellipsoid, fractional anisotropy (FA) is a commonly derived measurement. FA reflects the deviation of water motion within the voxel from that of the standard isotropic diffusion or free water. FA is calculated from the eigenvalues of the ellipsoid to provide a normalized value to the tensor’s degree of anisotropy (0 is completely isotropic, like free water, and 1 is completely anisotropic) [8]–[11], [15], [16]. Another DTI measure is diffusivity which includes Axial diffusivity (AD), radial diffusivity (RD) and mean diffusivity (MD), all calculated from the eigenvalues (λ) of the tensor ellipsoid. Those measurements reflect various forms of water diffusion within the ellipsoid: AD measures diffusion along the longitudinal axis of the ellipsoid (fastest diffusion), while RD is the average diffusion perpendicular to the axis of the ellipsoid. MD, is the average of all three eigenvalues, and is assumed to provide the apparent extent of water diffusion within a certain tissue per unit time (mm^2^/s). Alterations of MD and FA are usually thought to reflect microstructural changes in the tissues. Those changes include various pathologies such as axonal damage, demyelination, decreased axonal density, cellular death or proliferation, among others. Differential changes in AD and RD have been sometimes described in specific pathologies, such as increased RD in demyelination [17]–[19] and decreased AD in association with axonal damage [20], [21]. While DTI provides insight into tissue pathologies, it is often difficult to implicate a single pathological feature as the cause of FA and Diffusivity changes, and interpretation of those changes can vary between groups. In some cases, DTI changes have been related to obvious pathologies such as for example in aging where MD increases and FA decreases. In the case of aging, the changes in DTI parameters are assumed to be due to increased spacing between cell membranes due to volume loss, thus decreasing the directionality and increasing the diffusivity of water molecules [11], [14], [22], [23].

Using structural MR in HIV+ patients, it was almost unanimously found that dramatic brain volume loss occurred in the pre-ART era [24], [25], became less severe, but did not completely resolve in the post-ART era [5], [26]. On the other hand, DTI results in HIV+ patients were less consistent across studies, although the majority reported decreased FA and increased MD (similar to aging effects) in various brain regions [6], [27]–[29]. Divergent DTI results in HIV+ patients could be partially due to the use of different imaging techniques by various research groups as well as different methods of analysis, region of interest selection, parameter calculation, and different assumptions while interpreting the results.

The HIV-1 Transgenic (Tg) rat is known to develop neurological abnormalities with age and has been proposed as a model of chronic HIV infection in the post-ART era [30]. Neurologic damage is assumed to result from chronic exposure to viral proteins which are known to induce neurotoxic effects [30]–[32]. In this study, in order to further understand the volumetric and DTI abnormalities reported in HIV+ patients, we used the HIV-1 Tg rat as a small animal model of HIV infection [31] and obtained cross-sectional and longitudinal volumetric and DTI MR imaging using a 7T scanner. Immunofluorescence staining was obtained in order to explain the MRI findings in a pathophysiological context of brain damage.

## Materials and Methods

### Animals

Adult male rats (Controls = Fisher 344/NHsd; Tg = HIV-1 transgenic rat model) were used in this study, with the approval of the NIH Animal Care and Use Committee (ACUC).

The MR imaging protocol included structural and diffusion tensor experiments that were collected on four cohorts of animals: Nineteen young animals (10 Tg, 9 Control, mean ages 3.2±0.3 and 2.8±0.4 months respectively) and 17 middle-aged animals (10 Tg, 7 Control, mean ages 9.7±1.2 and 9.6±0.7 months respectively). A subset of the middle-aged animals (7 Tg, 3 Control) was reimaged approximately six months later (mean ages 15.2±0.5 and 16.1±0.54 months, respectively) to determine if accelerated brain atrophy or changes in diffusion characteristics occurred with aging.

### Volumetric Imaging

Animals were anesthetized using isoflurane and imaged for a total of 2 hours on a Bruker Biospin Avance III 7T scanner (Paravision 5.1) with a 21 cm bore, using a Bruker-Biospin BGA12S 12 cm shielded gradient with 2nd order shims (Billerica, MA). Body temperature and respiration rate of the rats were continuously monitored during imaging. A cross coil setup was used with a 72 mm transmission coil and 20 mm reception surface coil placed on the animal’s head. All anatomical images were obtained using an NIH version of a 3D modified driven equilibrium Fourier transform (MDEFT) sequence for volumetric measurements [33]. For the 3 month old animals, images were obtained in the axial plane. Parameters included: Echo time (TE) of 4.4 ms, 2 segments, a segment repetition time (TR) of 3864 ms, slice thickness of 0.5 mm, field of view (FOV) of 35×35 mm, 16 averages, spatial resolution of 0.14×0.14 mm/pixel and matrix size of 256×256 (∼56 min). Images were post processed to a matrix of 512×512×64 resulting in a resolution of 0.07×0.07×0.2 mm. For the 9 and 15 month old animals, images were obtained in the coronal plane. Parameters included: TE of 4.4 ms, 2 segments, a segment TR of 3760 ms, slice thickness of 1 mm, FOV of 35×35 mm, 16 averages, spatial resolution of 0.18×0.15 mm/pixel and matrix size of 192×240 (∼56 min). Images were post-processed to a matrix of 512×512×64 resulting in a resolution of 0.07×0.07×0.44 mm. The NIH open source software, medical image processing, analysis and visualization (MIPAV) [34] was used for manual masking and volume of interest (VOI) placements to allow for measurement of the total brain volume (TBV), ventricular volume (VV) and parenchymal volume (PV = TBV-VV).

### Diffusion Tensor Imaging

**Figure 1:**
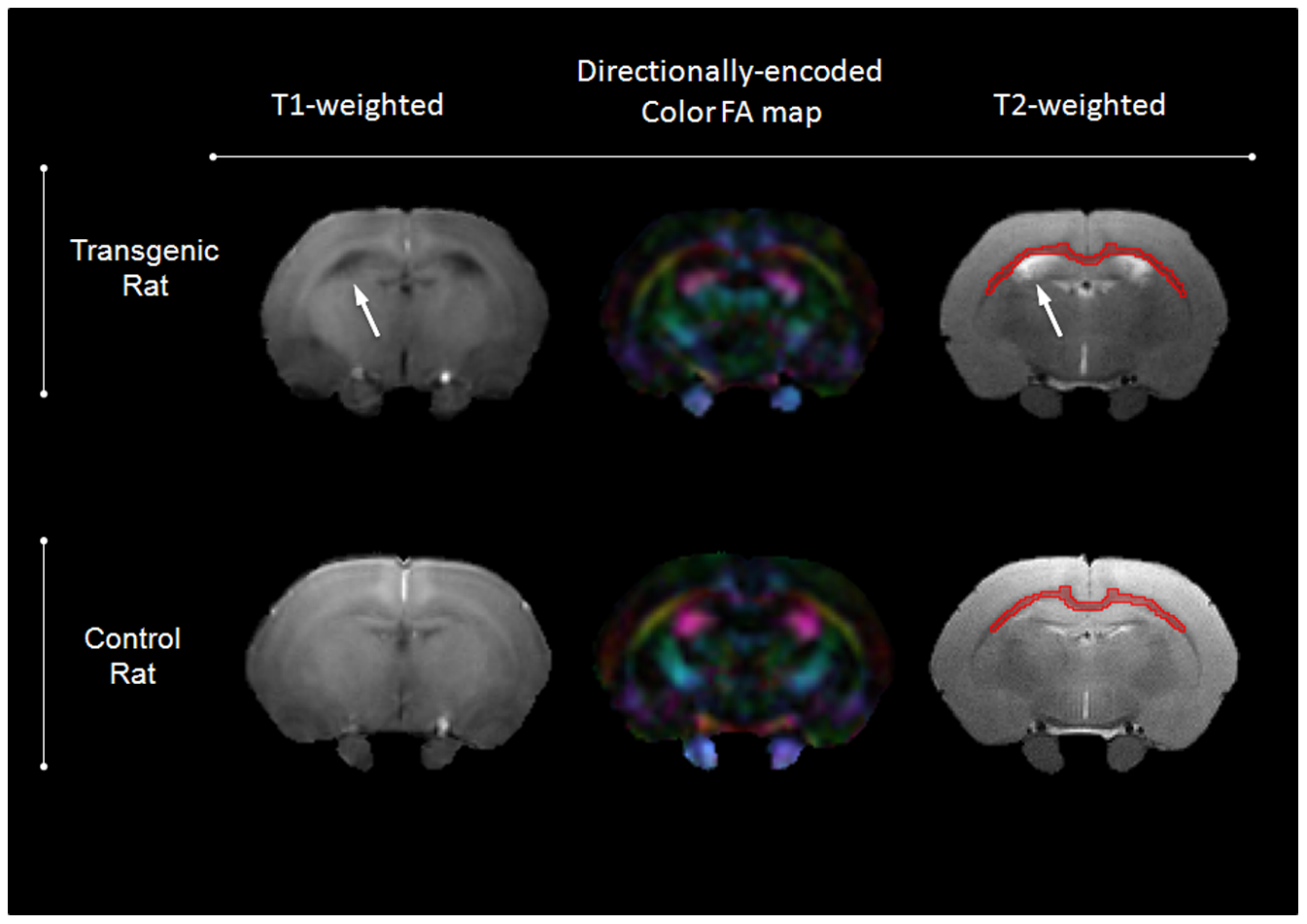
Coronal T1-weighted, directionally-encoded color FA map and T2-weighted MR images obtained from middle-aged transgenic rat (A) and age-matched control rat (B). White arrows point to enlarged ventricular size in the Tg rat. The red contour delineates the ROI selected for measurement of DTI parameters in the CC.

Utilizing the same coil configuration as above, an echo-planar imaging based DTI sequence was used. Parameters included: 0.27×0.27 mm/pixel spatial resolution, 128×128 matrix size, TE of 29.40 ms, TR of 8000 ms, 2 averages, 12 diffusion directions, and 1 mm slice thickness (∼15 minutes). The highest b value used was 1000 s/mm^2^. T2-weighted images were acquired (rapid acquisition with refocused echoes, RARE) in the coronal plane for image co-registration and distortion/motion correction of the diffusion-weighted images (Figure. 1). Parameters included: 0.14×0.14 mm/pixel spatial resolution, 256×256 matrix size, 8 averages, RARE factor of 8, TE of 12.3 ms, TR of 3379 ms, and 1 mm slice thickness (∼15 minutes).The NIH open source program TORTOISE was used for EPI distortion, motion, and B0 correction [35]. MIPAV was used for region of interest (ROI) placement in the corpus callosum (CC). The CC ROI corpus callosum is the average of 3 ROIs taken from 3 consecutive 1 mm-thick coronal slices (Figure. 1), situated approximately between Bregma +0.6 mm and Bregma −1.6 mm. This was done by comparing the T2 images to the Paxinos atlas [36].

### Immunofluorescence

Rats were first anesthetized with Isoflurane (3% with 700 cc/min O2). This was followed by transcardial perfusion using 100 ml of normal saline (pH = 7.4) and 350 ml of freshly prepared and filtered (0.45 micron filter) 4% paraformaldehyde (pH = 7.4). Brains were removed and post-fixed overnight in 4% paraformaldehyde at 4°C followed by three one-hour washes in normal saline at 4°C. Brains were next cryoprotected in 10% sucrose and stored at 4°C until they sank in the solution; they were subsequently placed in 20% and then 30% sucrose until they sank again in each solution. The brains were then embedded in optimal cutting temperature compound (O.C.T., Tissue-Tek) and ten-micron thick coronal serial sections were obtained. Multiepitope immunolabeling protocols were applied to identify the cellular phenotypes in fresh frozen brain slices using different combinations of primary antibodies. The antibodies relevant to this manuscript included guinea pig IgG anti-NeuN (EMD Millipore, cat# ABN90P) to identify most neurons, mouse IgG2b anti-GFAP (BD Biosciences, cat# 556330) to identify astrocytes, and rat IgG anti -Neurofilament H (EMD Millipore, cat# MAB5448) to label neuronal processes. Each of the above primary immunoreactions was visualized using appropriate fluorophore-conjugated (Alexa Fluor dyes) secondary antibodies obtained either from Jackson ImmunoResearch (West Grove, PA) or Life Technologies/Invitrogen (Carlsbad, CA). The cell nuclei were counterstained using 1 ug/ml DAPI to facilitate cell counting. All fluorescence signals were imaged using an Axio Imager.Z2 upright scanning wide field fluorescence microscope (Zeiss, Oberkochen, Germany) equipped with Orca Flash 4.0 high resolution sCMOS camera (Hamamatsu Photonics, Hamamatsu, Japan), 200W X-cite 200DC broadband light source (Lumen Dynamics, Mississauga, Canada) and standard DAPI and various Alexa Fluor filter sets (Semrock, Rochester, New York). After imaging, the multichannel image datasets were processed for image stitching, illumination correction and the images imported into Adobe Photoshop CS6 to produce pseudo-colored multi-channel composites.

Quantification of immunofluorescent staining was performed using FIJI image processing package, based on ImageJ (NIH). The locations of the selected striatal and cortical ROIs (each ROI measured 0.06 mm2) were identical between all the animals. For Neu-N cell counts, the RGB bitmap images were first converted to 8-bit grayscale and the threshold was adjusted to include only cells of interest and eliminate the background, and this was followed by counting using ITCN plugin. All images were processed using the same analysis parameters. The cell density (cells/mm^2^) was calculated from the total number of positive cells divided by the total area. For the staining intensity measurements of GFAP and NFH, the background fluorescent signal was removed by a thresholding process, visually selected by the user. The same cutoff value was then used to analyze all the slides stained at the same time. In addition to striatal and cortical ROIs, three ROIs were chosen within the corpus callosum (central, right and left).

The striatal sections from 8 young animals (3 month-old; 5 Tg and 3 controls) and 5 middle-aged animals (7 month-old; 3 Tg and 2 controls) were used for the analysis. After the absolute Neu-N counts and absolute GFAP and NFH intensities were calculated for all animals, each value from a Tg animal was divided by the value form the control rat(s) stained in the same session. This yielded 5 ratio values for the young cohort and 3 ratio values for the middle-aged cohort, except for NFH where we had only two values for the middle aged animals. The means of the ratios are shown in Table. 1.

**Table 1:** Quantitative measurements of Neu-N counts and GFAP and NFH staining intensities in the young and middle-aged animals, in various locations.

	ROI	3 month-old (n = 5)	7 month-old (n = 3)
**Neu-N**	**Cortex**	0.736±0.09	0.749±0.15
	**Striatum**	0.471±0.15	0.570±0.3
**GFAP**	**Cortex**	0.374±0.18	0.318±0.13
	**Striatum**	0.445±0.15	0.223±0.13
	**Corpus callosum**	0.565±0.25	0.230±0.004
NFH*	**Cortex**	0.874±0.11	0.934±0.04
	**Striatum**	0.671±0.26	0.935±0.29
	**Corpus callosum**	0.85±0.26	0.622±0.13

The numerical values represent the mean and standard deviations of staining count ratios (for Neu-N) and staining intensity ratios (for GFAP and NFH) of Tg/Control animals. Only animals stained in the same session were compared and used to obtain the ratios. Data was obtained from 8 young animals (5 Tg and 3 controls; 5 ratios) and 5 middle-aged animals (3 Tg and 2 controls; 3 ratios) except for NFH (*) where we had only two ratio values for the 7-month old group.

### Statistics

For the cross-sectional imaging study, two-tailed Student’s t-test (equal variance assumed) was used to assess for differences (*p*<0.05) between cohorts only if the analysis of variance (ANOVA) over all cohorts was deemed significant (*p*<0.05) or suggested a trend (*p*<0.10).

In one instance (VV measurements), we noticed that the data distribution in one of the groups (middle-aged Tg) might not be normal. To test for normality, we obtained a Q/Q plot and a histogram (data not shown) which confirmed that the sample was not normally distributed. Due to the lack of normality of distribution in the ventricular size in one of the subgroups, we decided to use the Kruskal-Wallis ANOVA test to assess for significance rather than the regular ANOVA. This would be followed by non-parametric Wilcoxon two-sample test to compare differences in-between groups if the Kruskal-Wallis ANOVA shows significant differences (p<0.05).

For the longitudinal imaging study, significant changes over time were determined using matched pair t-tests for both the Tg and control cohort.

## Results

### Cross-Sectional Volumetric Imaging

**Figure 2:**
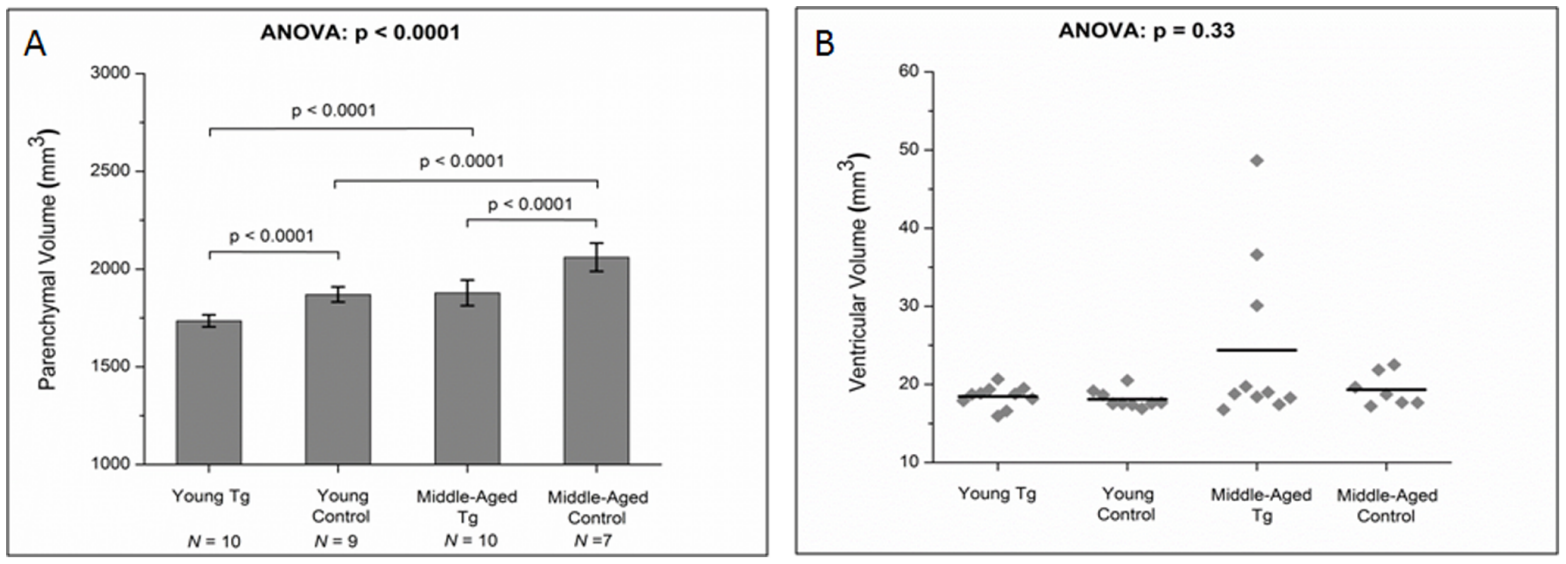
Changes in (A) parenchymal volume and (B) ventricular volume, between the Tg rats and their age-matched controls at both young and middle-age.

We found that both young and middle-aged Tg animals had significantly lower TBV (ANOVA, *p*<0.0001) and PV (ANOVA, *p*<0.0001) compared to their corresponding age-matched controls (Figure. 2A). Tg rat brains were ∼7% smaller than corresponding controls in the young animals group and ∼8.5% smaller in the middle-aged animals. TBV and PV were significantly greater in the middle-aged animals compared to the young animals, both in the control (∼10%, *p*<0.0001) and Tg groups (∼8%, *p*<0.0001) suggesting incomplete brain development in the young animals. Qualitatively, the ventricles were comparable between the Tg and control groups at young age, but 30% of the middle-aged Tg cohort showed visually noticeable ventricular enlargement (Figures. 1 and 2B). Due to the lack of normal distribution in the middle-aged Tg group (shown by Q/Q plot and histogram), we used the Kruskal-Wallis ANOVA test which resulted in p value of 0.33 indicating lack of significance.

### Cross-sectional DTI imaging results

**Figure 3:**
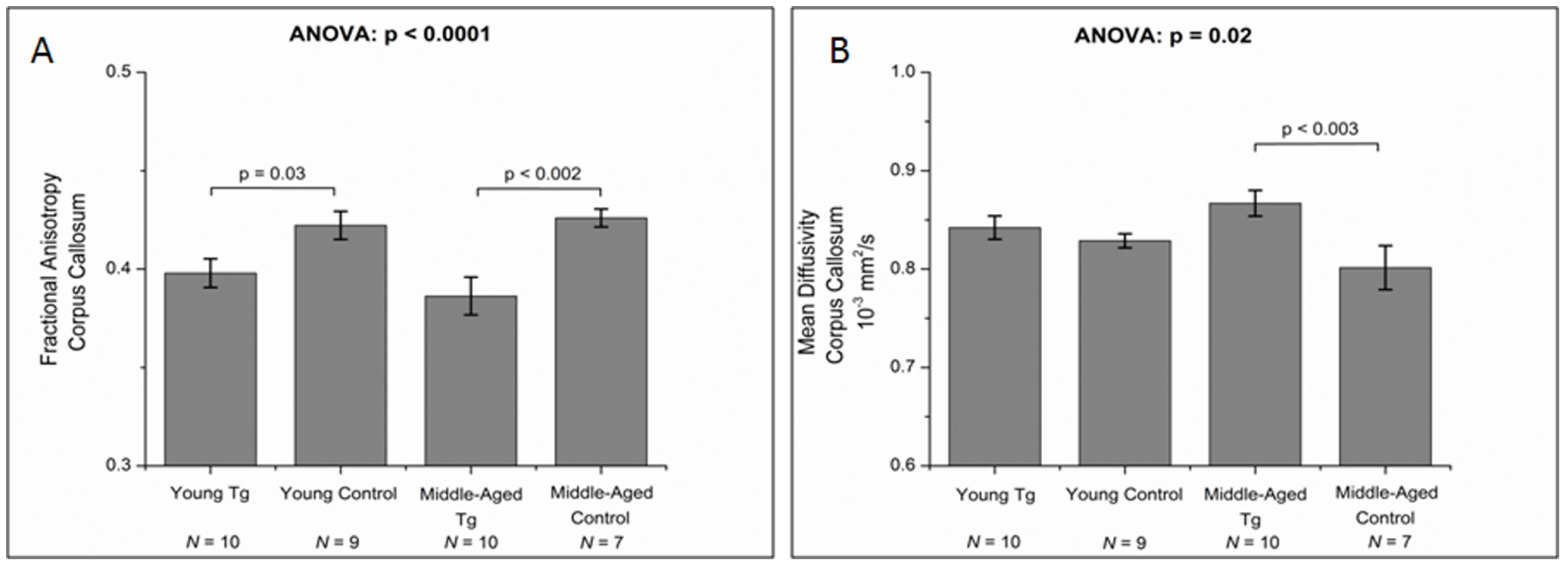
Changes in (A) CC fractional anisotropy and (B) CC mean diffusivity between the Tg rats and their age-matched controls at both young and middle-age.

Statistically significant lower FA values were seen in the CC of both young and middle-aged Tg cohorts in comparison to their corresponding age-matched control groups (*p* = 0.03 and *p*<0.002 respectively) (Figure. 3A). MD values in the CC were higher in the Tg rats when compared to the corresponding controls, but this was only significant in the middle-aged animals (*p*<0.003) (Figure. 3B). Those changes in MD values were primarily driven by changes in RD rather than AD. No significant differences in CC MD values were observed between the young and middle-aged Tg or between the young and middle-aged control rats (Figure. 3B).

### Longitudinal Volumetric Imaging Results

In the subset of middle-aged animals (7 Tg and 3 controls) that were re-imaged approximately 6 months after initial imaging, we found reductions in brain volume in the Tg rats but those reductions did not reach statistical significance (TBV: *p* = 0.23, PV: *p* = 0.16). TBV and PV within the 3 control rat brains remained fairly constant between the first and second imaging sessions (Table. 2). VV significantly increased in size over time in the Tg rats (+19%, paired t-test: *p* = 0.005) but not in the controls (Table. 2). Out of the three animals with significantly larger ventricles in the cross-sectional middle-aged group, only one was included in the longitudinal study. Irrespective of the VV at 9-months of age, however, all Tg animals experienced a similar pattern of enlarging ventricles over time.

**Table 2:** Summary and Statistical Analysis of MR Measures in the Longitudinal Study.

Measure	Tg rats^1^		PairwiseT-test TgGroups (*p*-value)	Control rats^1^		Pairwise T-testControlGroups (*p*-value)
	9–10month old	15–16month old		9–10monthold	15–16 monthold	
**Brain Volume (in µl)**						
Total Brain (TBV)	1918±64	1896±85	N.S.	2076±86	2118±25	N.S.
Parenchyma (PV)	1897±63	1871±85	N.S.	2058±87	2098±23	N.S.
Ventricles (VV)	21.2±6.8	25.3±8.6	**0.005**	18.7±1.0	19.9±1.6	N.S.
**Corpus Callosum DTI**						
Fractional Anisotropy (FA)	0.38±0.03	0.40±0.01	N.S.	0.43±0.01	0.43±0.02	N.S.
Mean Diffusivity (MD)	0.86±0.03	0.80±0.04	**<0.03**	0.80±0.06	0.81±0.01	N.S.
Axial Diffusivity (AD)	1.27±0.13	1.20±0.14	**<0.04**	1.19±0.07	1.20±0.05	N.S.
Radial Diffusivity (RD)	0.70±0.06	0.65±0.06	**<0.04**	0.61±0.05	0.60±0.04	N.S.

1 Values represent Average ± Standard Deviation of the 7 Tg and 3 control animals from the middle-age cohort that underwent repeated measures. MD, AD and RD units are×10^−3 ^mm^2^/s. N.S. = Not significant.

### Longitudinal DTI imaging results

No significant changes in FA values were found for either the control or Tg rat cohorts in the longitudinal study. Significant decreases in MD, RD and AD values were however seen within the Tg rat population over the 6 months follow-up period (Table. 2). AD and RD appear to be equally contributing to MD changes over time. No changes in MD, RD and AD values were seen over time within the control group.

### Immunofluorescence staining results

On immunofluorescence staining of the striatum and cortex from two age groups (young and middle-aged animals), there were decreased Neu-N counts in the Tg compared to the age-matched control animals (Table. 1). The Neu-N decrease was more significant in the striatum than the cortex in both age groups which could potentially explain the central pattern of volume loss that was seen in Tg animals. In addition, significant astrocytic loss/dysfunction (decreased GFAP staining intensity) was noted in the Tg animals in both groups, although those changes were more significant in the middle-aged group, namely in the striatum (Table. 1). Decreased NFH staining suggesting axonal damage/loss was also noted. This however did not follow the same trend of worsening with age as the other markers. This could possibly be due to a small sample number (n = 2) in the middle-aged group since one of the control slides was deemed suboptimal for NFH staining and was excluded. Astrocytic and axonal damage/loss probably contribute to the total brain volume loss.

**Figure 4:**
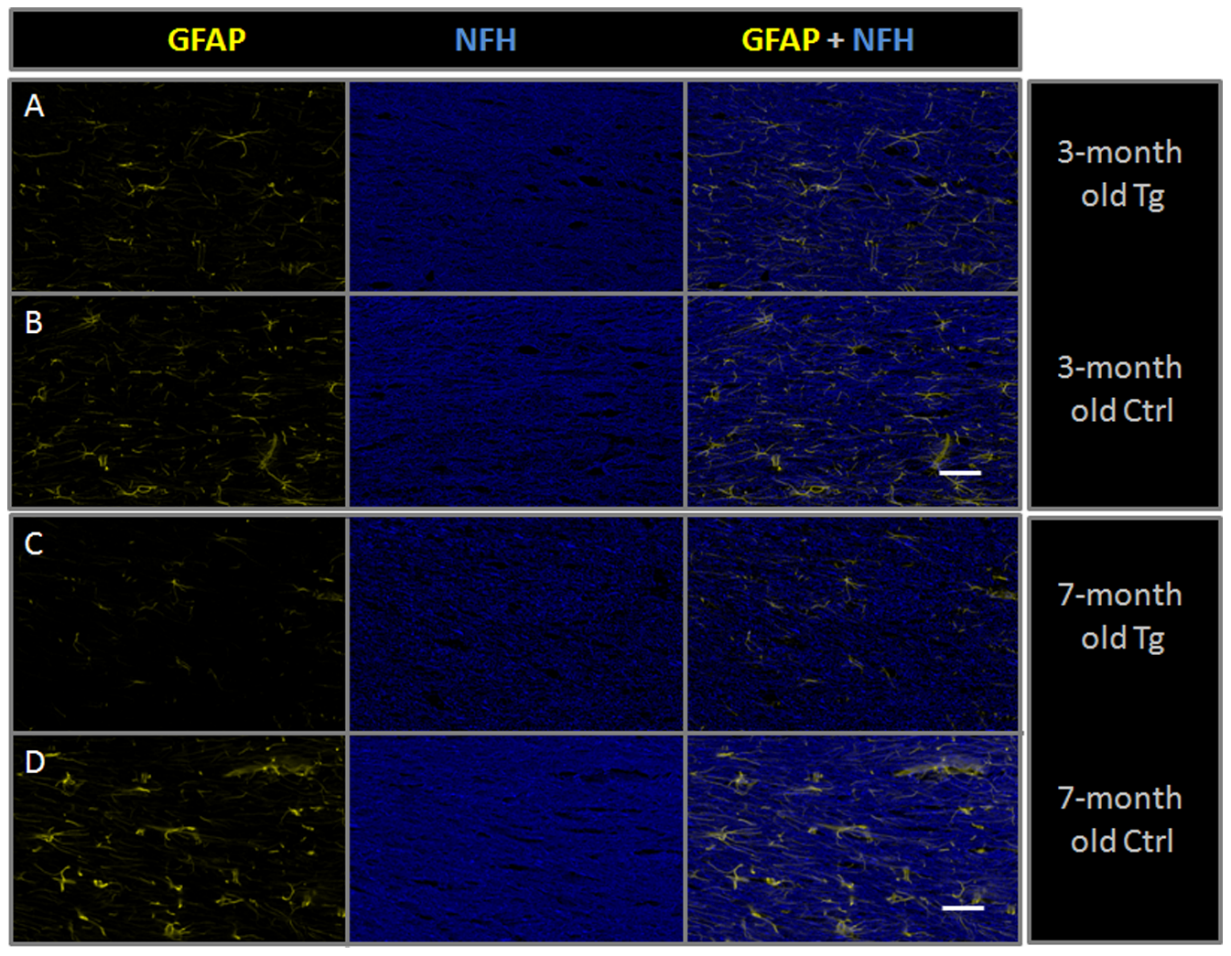
Immunofluorescence images from age-matched 3-month old Tg (A) and control (B) rats and from age-matched 7-month old Tg (C) and control (D) rats. The captures were obtained from the central portion of the corpus callosum in each animal. Each row shows GFAP staining in yellow, NFH staining in Blue and a composite image of both (magnification 100X, scale bar = 40 µm). Subtle decreased GFAP and NFH staining are seen in the young Tg compared to the young control rat, however those differences become more noticeable in the 7-month old Tg compared to the age-matched control.

In the CC region, GFAP staining was also decreased in the Tg rats compared to age-matched controls in the young cohort (mean ratio of staining of Tg compared to control = 0.565). This seemed to worsen with age (mean ratio of staining in middle aged animals = 0.229). NFH intensity of staining in the Tg CC was also decreased compared to controls and worsened slightly with age (Figure 4 and Table. 1).

## Discussion

The persistence of HIV-associated neurocognitive disorders (HAND) despite successful treatment of HIV infection in the periphery has necessitated a new approach that emphasizes neuroprotection rather than eradication of the virus from the brain, as the latter has proven unsuccessful until now. Multiple molecules have been evaluated or are still being suggested as potential neuroprotective therapies for HAND [37]–[43]. The field however lacks two important elements that would allow efficient screening of protective molecules/techniques: 1. an appropriate and practical animal model and 2. reproducible *in vivo* biomarkers of diseases. The SIV infected monkey is an excellent model for HIV; however it is expensive to acquire, house and sustain, especially when large sample numbers are needed [44]. In this study, we wanted to evaluate the HIV-1 Tg rat as an appropriate model for neuro-HIV and establish reliable MR-based *in vivo* biomarkers that could be used to assess the effectiveness of various neuroprotective approaches within this model.

The HIV-1 Tg rat, which we chose as our model, is commonly used, readily commercially available, and non-infectious [31]. It also presents with measurable cognitive deficits [32], and has been recently proposed as a potential model for treated HIV+ patients [30], considering the lack of viral replication and the prolonged exposure to viral proteins such as Gp120, tat and nef [45]. We pursued volumetric imaging of this animal model based on brain volume loss being one of the earliest recognized abnormalities in HIV+ patients [24], [25]. This volume loss persisted in the post ART era with white matter and basal ganglia volume loss still demonstrable in virologically suppressed patients [5], [46], [47]. We also used DTI since this technique has been applied extensively in the evaluation of HIV+ patients, often with positive although not always consistent results [7], [28], [48]–[50]: generally decreased FA and increased MD [14], [22], [23].

Using volumetric imaging, we found reduced brain volumes in young and middle-aged Tg rats compared to their age-matched controls, consistent with published human findings [25]. We also found larger ventricular size in a subset of middle-aged Tg compared to young Tg animals (Figure. 2B) while no such finding was depicted in the controls. A stronger pattern emerged in the longitudinal component of our study where Tg VV significantly increased in the Tg animals while no changes were observed in the control rats (Table. 2). Interestingly, PV and TBV of the Tg animals did not change significantly over time (Table. 2).

On DTI, FA values were lower and MD values were higher in the CC of the Tg rats compared to controls in the middle-aged cohort. Those findings might be partially explained by the pathology depicted on immunofluorescence including astrocytic loss/dysfunction and axonal damage (Figure. 4 A-D) however a direct causal relationship cannot be ascertained. The high MD values (Figure. 3B) and decreased FA values (Figure. 3A) in this case are similar to what is seen with aging [22], [23], assumingly for similar reasons (cellular and axonal loss resulting in increased spacing between cell membranes due to volume loss and secondary decreased directionality and increased diffusivity of water molecules). Interestingly, we noticed that the changes in MD are mainly driven by changes in RD which is in concordance with previous observations that eigenvalue amplitudes (λ) or combinations of the eigenvalues, such as RD (λ_2_+λ_3_)/2, can be more specific to white matter pathology than AD [17]. The findings in the CC are consistent with published human DTI results from the post-ART era [6], [28], [29], [48]–[50]. Our findings in the CC are also consistent with changes reported in treated HIV+ subjects (higher MD in the CC) compared to untreated HIV+ individuals (lower MD) [47], further supporting the concept that the Tg rat might be a good model for treated HIV+ patients.

We did not see significant changes in CC FA values for either the control or Tg rat cohorts in the longitudinal study (Table. 2), which in the Tg rat could reflect either lack of sensitivity of the technique to minor changes, or a maximum degree of damage to the brain around 9–10 months of age that would not be significantly worse at 15 months of age (burnt-out). There was however a drop rather than increase in diffusivity values in the Tg animals’ CC over time. Interestingly, unlike in the middle-age cohort of animals where the difference in MD between Tg and controls was mainly driven by change in RD, a measure linked to myelin integrity, the change in MD with aging in the Tg rats was equally driven by RD, as well as AD, a measure linked to axonal integrity [51]. A disproportional loss of axonal integrity with aging could explain the MD changes in the older Tg rats. This however needs to be further investigated.

There are a few limitations to our study. First, the HIV-1 Tg rat is not a natural infectious model like the SIV-infected monkey since multiple constraints prevent active viral replication in rodents [31], [52]. It remains however one of the best small animal models reflecting neuro-HIV pathology, especially with behavioral abnormalities documented by many groups [31], [32], [53], [54]. It is also a non-infectious model, which significantly increases the ease of using it in various settings. Another limitation is the small number of animals in the longitudinal study, especially the control group, and the lack of longitudinal data in the younger animal groups. Finally, we are bound by the inherent limitations of DTI as an imaging technique. Perhaps the most important of those limitations is the unavoidable averaging of crossing fibers with differing directions within the same voxel [55]. The choice of the CC helps limit this problem since the fibers are well packed and oriented parallel to each other. Another technical limitation is the susceptibility of DTI to motion, which we tried to overcome by maintaining a stable anesthesia plane throughout the scan acquisition time as well as with post processing motion correction using various programs.

While the exact pathophysiology underlying volumetric and DTI changes in the Tg rat needs to be further examined, we hypothesize, based on immunostaining, that it could be related to glial (astrocytic) cellular death, neuronal dysfunction/loss and axonal damage/loss, all likely related to prolonged exposure to HIV viral proteins, namely gp120, nef and tat. Indeed, HIV proteins’ mRNA expression is known to increase in the Tg rat brain (cerebellum and spinal cord) with age [30]. Our results from immunostaining are also consistent with prior reports suggesting that the neurologic dysfunction seen in HIV brains correlates better with dendritic loss [56] than with neuronal apoptosis [57], [58].

## Conclusion

We believe that volumetric imaging in the HIV-1 Tg rat can be used as a reliable imaging biomarker for brain damage in the prospective evaluation of HIV neuroprotective therapies: the ability of various interventions/therapies to halt volume loss in this animal model would constitute compelling preclinical data, driving further translation into human application.

DTI measures, despite being quite promising, still need further validation before they can be used as accurate and reproducible biomarkers of neurodegeneration.
